# Acoustic Emission Source Location Using a Distributed Feedback Fiber Laser Rosette

**DOI:** 10.3390/s131014041

**Published:** 2013-10-17

**Authors:** Wenzhu Huang, Wentao Zhang, Fang Li

**Affiliations:** Optoelectronic System Laboratory, Institute of Semiconductors, Chinese Academy of Sciences, Beijing 100083, China; E-Mails: hwzhu@semi.ac.cn (W.H.); lifang@semi.ac.cn (F.L.)

**Keywords:** acoustic emission, DFB fiber laser rosette, directional sensitivity, autocorrelation, wavelet packet energy, location algorithm

## Abstract

This paper proposes an approach for acoustic emission (AE) source localization in a large marble stone using distributed feedback (DFB) fiber lasers. The aim of this study is to detect damage in structures such as those found in civil applications. The directional sensitivity of DFB fiber laser is investigated by calculating location coefficient using a method of digital signal analysis. In this, autocorrelation is used to extract the location coefficient from the periodic AE signal and wavelet packet energy is calculated to get the location coefficient of a burst AE source. Normalization is processed to eliminate the influence of distance and intensity of AE source. Then a new location algorithm based on the location coefficient is presented and tested to determine the location of AE source using a Delta (Δ) DFB fiber laser rosette configuration. The advantage of the proposed algorithm over the traditional methods based on fiber Bragg Grating (FBG) include the capability of: having higher strain resolution for AE detection and taking into account two different types of AE source for location.

## 1. Introduction

Nowadays, acoustic emission (AE) detection is being widely used for non-destructive testing of concrete structures, such as bridges and viaducts or masonry historical buildings [1,2]. This method can predict the damage evolution and the time to structural collapse. Most traditional AE sensors utilized consist of piezoelectric elements undergoing transduction, which are commercially available [3], but these conventional sensors are either not capable or flexible enough for making the appropriate measurements in the case of electromagnetic interference environment or remote monitoring applications [4]. A number of fiber optic acoustic emission sensors suitable for these applications have been developed in the past 30 years. The majority of these sensors, such as the Mach-Zahnder [5,6], Michelson [7], Sagnac [8], or Fabry-Perot [9,10] detectors, are based on fiber optic interferometry. These sensors have considerable advantages over conventional piezoelectric sensors, such as immunity to electromagnetic interference and feasibility for long-term and long-distance structural condition monitoring, but they are pretty large in size and are difficult to multiplex.

In the past 10 years, FBGs have been successfully used for AE sensing applications [11–18]. FBGs offer distinct advantages for remote sensing such as ease of multiplexing, and simultaneous measurement of several parameters such as temperature and strain. FBGs are small in size and light in weight and can be embedded into material structure. Above all their directional responses make them suitable for determining principal strains and the direction of AE source [19]. However, in some cases, due to the limited sensitivity, FBGs can't detect the ultra-slight waves of the structure, and higher strain sensitivity is necessary. Recently, the new generation of optical fiber sensor based on distributed feedback (DFB) fiber lasers has received considerable research interest in this field [20]. The DFB fiber laser sensor has the advantages of small dimensions, ultra-narrow line-width, and low noise properties. It can reach ultra-high sensitivity in the detection of weak signals with high resolution wavelength demodulation technique. Acoustic emission detection using DFB fiber lasers offers not only the advantages associated with FBG sensors, but also better performance for ultra-slight acoustic emission [21].

In our previous work, we reported the realization of DFB fiber laser and DFB fiber laser rosette for acoustic emission detection [22,23]. The acoustic emission directional characteristics of the DFB fiber laser are demonstrated in experiments. According to our analysis on location method based on the directional sensitivity of the DFB fiber laser, the location precision is mainly limited by the intensity of AE source with high noise level. The previous location method will fail if the source strength changes.

This paper focuses on the use of DFB fiber laser rosette as an acoustic emission receiver. The principle of AE detection using DFB fiber laser is presented. A method of digital signal analysis, which can improve Signal to Noise Ratio (SNR) is used to investigate the directional sensitivity of the DFB fiber laser. And the normalization method is processed to eliminate the influence of distance and intensity of AE source. Then we configured three DFB fiber lasers into a rosette for acoustic emission source detection and location based on a new location algorithm. The test results are given.

## 2. Operation Principles

A DFB Fiber laser consists of a length of Er^3+^-doped or Yb^3+^/Er^3+^-doped fiber with Bragg gratings. By introducing a π phase shift, the grating resonance is moved to the center of the grating reflection band, which makes DFB fiber laser operate robustly in a single longitudinal mode. In our configuration, a phase-shifted grating is formed into a length of Er^3+^ fiber, whose ends are spliced to a matching passive fiber for reducing splice loss. The grating is pumped with a 980 nm or 1,480 nm semiconductor laser. The wavelength of DFB fiber laser is determined by the central wavelength in the reflective spectrum of phase-shifted grating, which is shown as:

(1) $$\lambda_{\text{B}}=2n_{\text{eff}}\Lambda$$

where *λ_B_* is the lasing wavelength, *Λ* is the period of grating, and *n_eff_* is the effective index of fiber core. The period *Λ* and the effective reflective index *n_eff_* are changed with the environmental conditions, such as strain, temperature, and acoustic wave.

**Figure 1.**
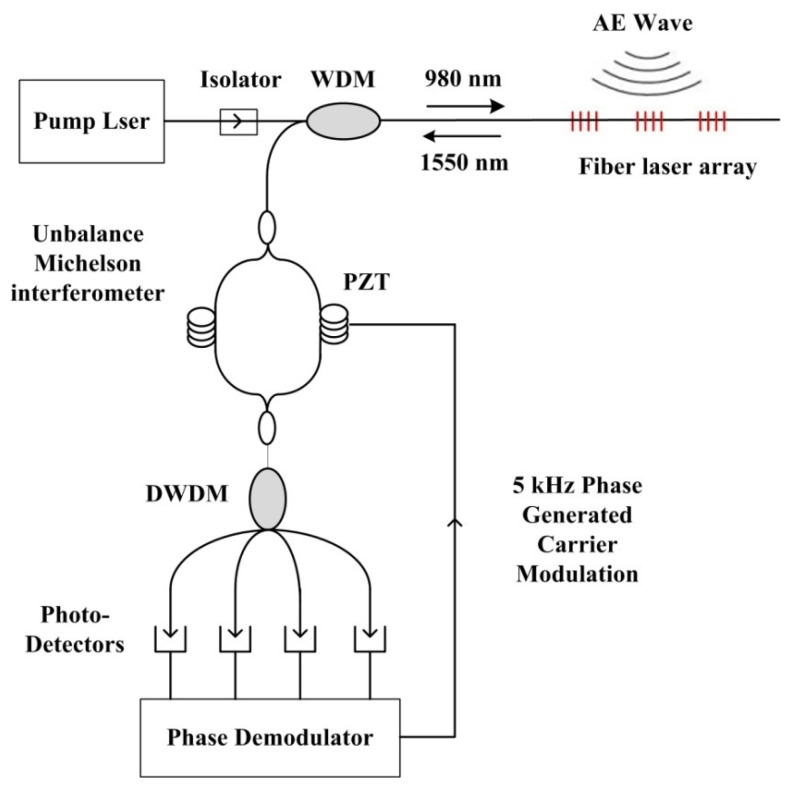
The demodulation diagram of DFB fiber lasers.

Figure 1 shows the schematic of a digital phase generated carrier (PGC) based wavelength demodulation system for DFB fiber laser sensors. The PGC scheme is used in the system to recover the phase signal and overcome the bias-drift-induced fading. One arm of the interferometer is wrapped onto a PZT tube and the phase modulation of the PGC scheme is introduced to the interferometer by electrically modulating the PZT. The interferometric signals are then received by a photo-detector and an amplifier. The electrical output of the amplifier is digitalized in accordance with the carrier signal using an A/D convertor. The PGC phase demodulation is accomplished on a FPGA board or in a computer.

**Figure 2.**
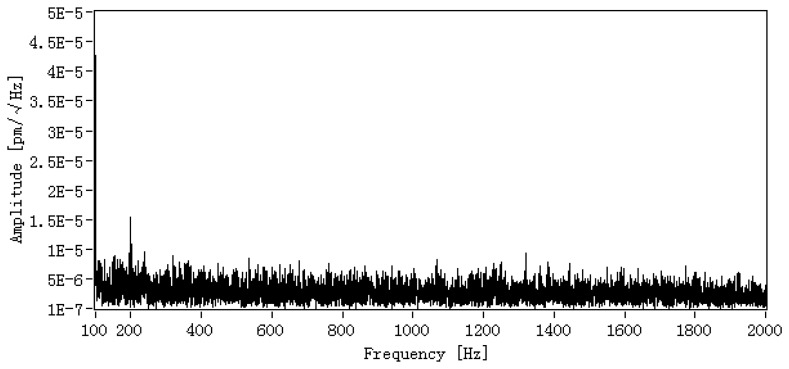
Noise level of DFB fiber laser sensing system.

The strain resolution of DFB fiber laser acoustic sensor is limited by system noise level, which is determined by frequency noise and relative intensity noise (RIN) of DFB fiber laser, electrical circuit noise, and environmental noise on the interferometer. The noise level is measured in a quiet laboratory with the isolation of vibrations and acoustic noise. Figure 2 shows the noise level in the frequency range from 20 Hz to 2 kHz and it can be observed that the noise level is below 1 × 10^−6^ pm/√Hz@1 kHz, which will result in a strain resolution of about 10^−12^.

It has been proved that FBGs exhibit maximum sensitivity to acoustic waves when the direction of maximum strain is parallel to the fiber axis and minimum when it is normal to the axis [19]. The directional responses of FBGs make them suitable for determining principal strains and the direction of propagation of incident acoustic waves. In our research, DFB fiber lasers have shown a similar trend. Here, the directional responses of DFB fiber lasers, instead of FBGs, are analyzed when they are used as acoustic emission receivers.

## 3. Investigation of AE Directional Sensitivity of DFB Fiber Laser

The acoustic emission experiment is carried out in a large marble stone. The DFB fiber laser is glued to the marble plate and operate at different center wavelengths with 300 GHz (2.4 nm) spacing from 1,529 nm to 1,546 nm, and pumped through a single fiber by a 980 nm pump laser. The center wavelength of fiber laser will be modulated by acoustic waves produced by an acoustic emission source and was demodulated by the demodulation system. Generally, a PZT sensor is also glued to the marble plate close to the fiber laser as a reference.

Primarily, an effective coupled-mode should be selected to record the AE signal with maximum sensitivity in the experimental process, which will much more closely reflect the features of AE source. Firstly, DFB fiber lasers are bonding on the surface of the marble plate with three different coupled modes, such as transparent adhesive, 502 adhesive and 353ND fiber adhesive. A PZT AE sensor is used to generate continuous sine acoustic waves. Then the DFB fiber laser of every coupled mode is used to detect the sine wave. We can find that the response of the DFB fiber laser coupled with 353ND fiber adhesive is more prominent than the other two kinds of cases, which shows that the coupled mode of 353ND fiber adhesive has the maximum AE sensitivity. In fact, this indicates that the fiber lasers can be used to detect the AE signal with minimum distortion when the fiber is fixed using 353ND fiber adhesive, which is suit for AE experiment.

**Figure 3.**
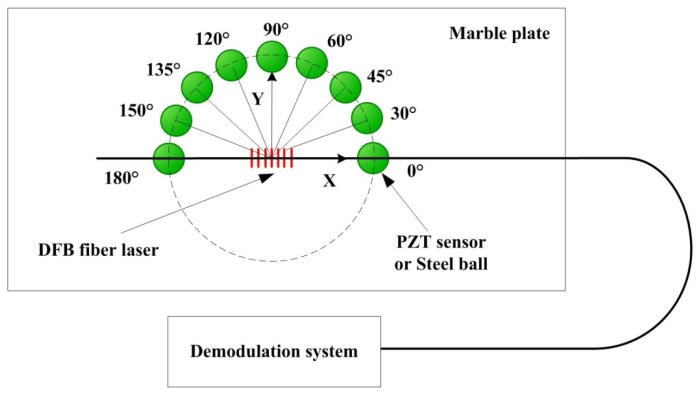
Investigation of the AE directivity of DFB fiber laser.

As we know, FBGs exhibit the directional responses and can be used to get the direction of the AE source [19]. In our research, DFB fiber lasers have shown a similar trend. The directional sensitivity of DFB fiber laser is investigated by calculating location coefficient using a method of digital signal analysis. A PZT sensor or steel ball is used as an exciting source respectively, which is shown in Figure 3.

As there are usually two kinds of AE source, one is a periodic AE source and another is a burst AE source, we should research a method to locate the different AE signal, so we use two kinds of digital signal analyzing method to investigate the directional sensitivity of DFB fiber laser and calculate the location coefficient for periodic AE source location and burst AE source location respectively. And a new location algorithm based on the location coefficient is proposed in the following.

**Figure 4.**
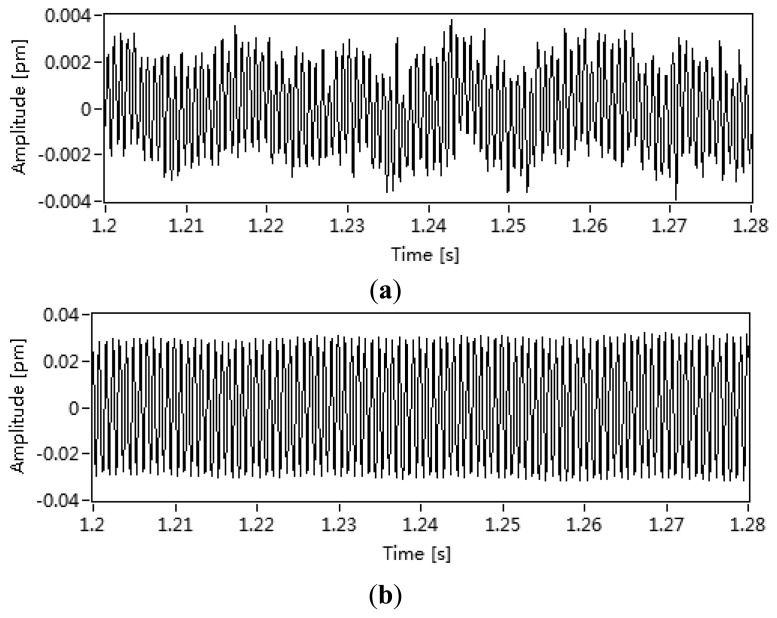
An autocorrelation approach is used to extract the periodic AE signal. (**a**) The amplitude of the signal detected by the DFB fiber laser and (**b**) the sine AE signal got by the autocorrelation method.

For a periodic AE source, the relationship between the wavelength drift of DFB fiber laser and the angle of the AE source is tested. The directivity of the DFB fiber laser has been tested by mounting PZT source transducers on the marble plate in a circular array with a DFB fiber laser at the centre. The amplitude of the signal detected by the DFB fiber laser was then measured as each PZT was excited in turn. Sine acoustic waves were generated by a PZT AE transducer (SR10-10112-B, Soundwel Technology Co. Ltd., Beijing, China), which was driven directly by a synthesized function generator. An autocorrelation approach is used to extract the periodic AE signal. We can get a sine AE wave with High SNR by the proposed method, which is shown in Figure 4. The autocorrelation is shown as:

(2) $${\text{R}}_{\text{xx}}(\tau )=\underset{T\to \infty }{\lim}\frac{1}{T}\int_{0}^{T}x(t)x(t+\tau )dt$$

where *R_xx_(τ)* is the result of autocorrelation, *x(t)* is the AE source, *T* is the time of sample data of the AE source.

**Figure 5.**
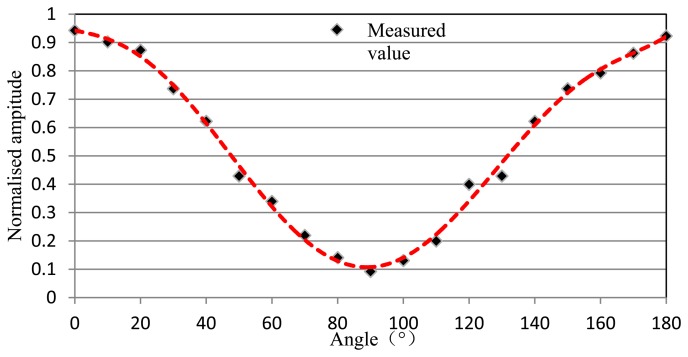
The relationship between wavelength drift and the angle of a periodic AE source.

In order to research the directional characteristic based on amplitude response of DFB fiber laser, amplitude normalization, which can eliminate the influence of distance and intensity of AE source, is processed. The relationship between the wavelength shift and the angle of an AE source is shown in Figure 5. Curve fitting shows a sine-squared relationship between the signal amplitude and the angle between the wave propagation direction and the DFB fiber laser axis. So we can get the direction of a periodic AE source according to the size of the amplitude response of DFB fiber laser. Linear normalization is shown as:

(3) $$\text{Nor}=\frac{\text{Act}-\text{Minvalue}}{\text{Maxvalue}-\text{Minvalue}}$$

where *Nor* is the value after the normalization, *Act* is the actual measurement value. *Maxvalue* is biggest sample value, *Minvalue* is minimum sample value.

**Figure 6.**
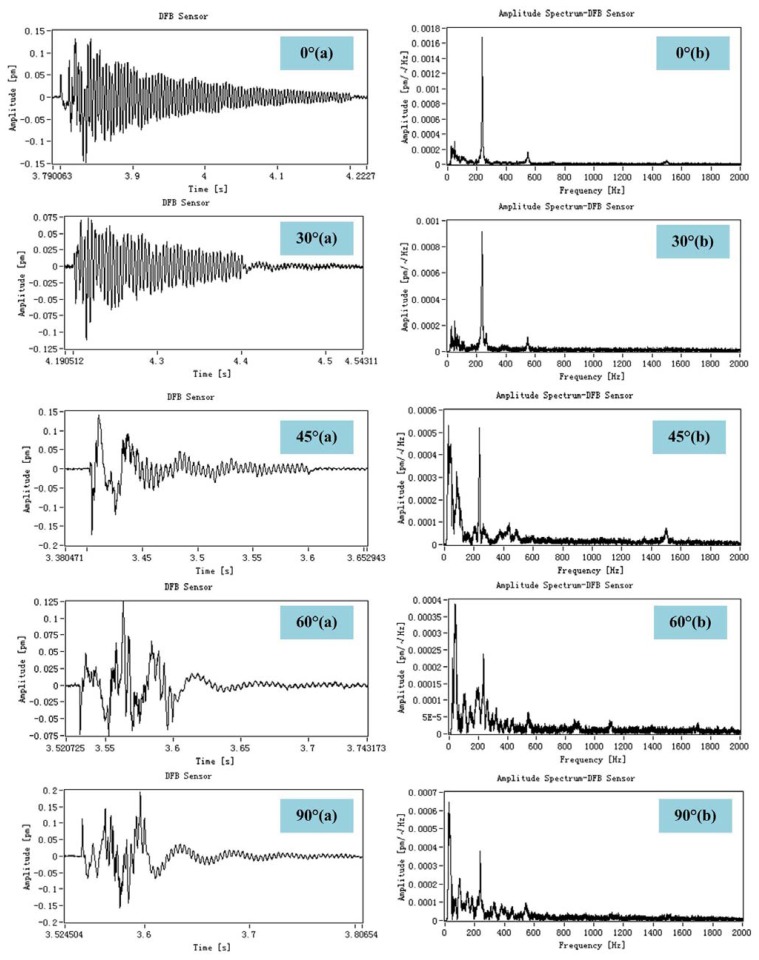
The relationship between percentage of wavelet packet energy of the response of DFB fiber laser and the angle of an impact response.0°(a), 30°(a), 45°(a), 60°(a) and 90°(a) represent the time-domain response of DFB fiber laser respectively. 0°(b), 30°(b), 45°(b), 60°(b) and 90°(b) represent the frequency-domain response of DFB fiber laser respectively.

For a burst AE source, the relationship between the percentage of wavelet packet energy of the response of DFB fiber laser and the angle of the impact response is established. Here, a steel ball with 9 mm diameter and 1.2 g in weight instead of PZT AE sensor is used to generate impact signal. The ball was dropped from a height of 20 cm above the horizontal marble plate. Then the impact response is measured by DFB fiber laser, which is shown in Figure 6.

We can see from the frequency-domain response of DFB fiber laser that the energy of dominant frequency around 200 Hz decrease and low-frequency component increase, which imply that we can get the direction of a burst AE source according to the size of the dominant-frequency component.

**Figure 7.**
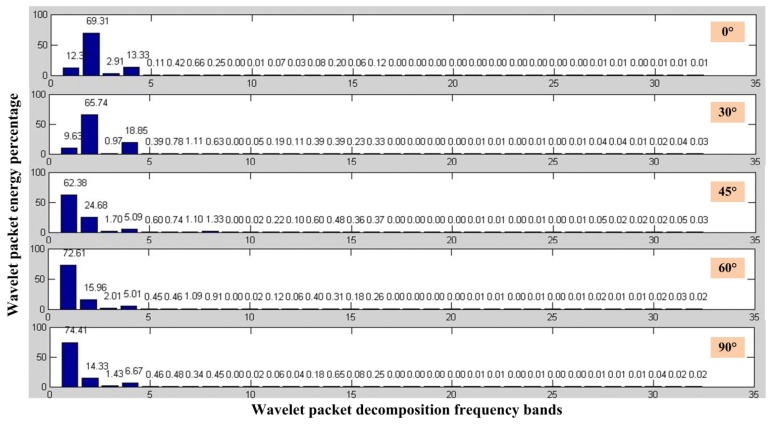
The wavelet packet energy percentage at different angle.

According to impact response we have got, we can calculate the wavelet packet energy percentage when the ball impact at different angle, which is shown in Figure 7. This method can also eliminate the influence of distance and intensity of AE source.

According to the above analysis, the dominant frequency of the impact response is about 200 Hz and the sampling frequency is 10 kHz here, so the layer of wavelet packet decomposition is chosen as five. The frequency of 200 Hz will be in the third layer. We can find that the percentage of wavelet packet energy in the third layer decreases when the angle between fiber lasers and the burst AE source increases, which imply that we can get the direction of a burst AE source by calculating the he percentage of wavelet packet energy of the source. It's worth noting that the dominant frequency of the impact response should be measured before using this method.

**Figure 8.**
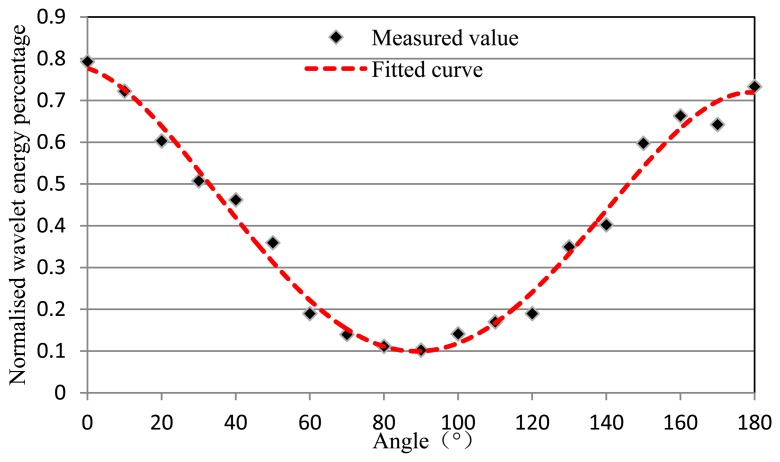
The relationship between percentage of wavelet packet energy and the angle of a burst AE source.

The relationship between percentage of wavelet packet energy and the angle of a burst AE source is shown in Figure 8. And it can be seen from the test results that the curve fitting is tub shape. According to the directional sensitivity of DFB fiber laser, we can use three DFB fiber lasers as a rosette which can determine the source location of an acoustic wave. Here, we can get the location coefficient of a periodic AE source from Figure 5 and get the location coefficient of a burst AE source from Figure 8. Then we can calculate the information for AE source location using an appropriate algorithm based on the location coefficient. This process is directly analogous to the use of ESG or FBG rosettes to determine principal strain magnitudes and directions.

## 4. Location Algorithm

**Figure 9.**
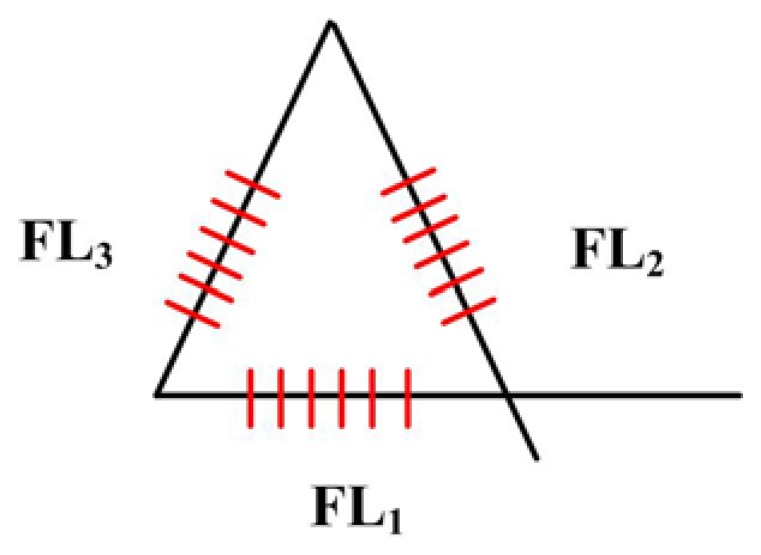
DFB fiber laser rosette.

There are many well-established configurations into which conventional electrical strain gauges (ESG) may be formed. One of these is the Delta (Δ) rosette configuration that is used to determine the direction and magnitude of the principal strain. Because DFB fiber lasers fulfill an equivalent function to their electrical counterparts, it seems appropriate to investigate the possibility of producing a DFB fiber laser rosette, which is shown in Figure 9. The DFB fiber lasers with different center wavelengths is the same piece of fiber, while a fourth may be added to provide either temperature measurement or compensation. The purpose of configuring DFB fiber lasers into a rosette is to detect and locate the source of an acoustic wave based on their directional characteristics of strain response.

**Figure 10.**
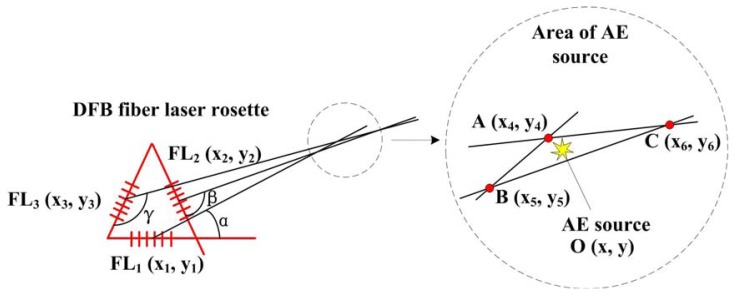
The principle of triangulation approach for AE source location based on DFB fiber laser rosette.

The principle diagram of triangulation approach for AE source location based on DFB fiber laser rosette is shown in Figure 10. Firstly, we can get the direction of the AE source through each fiber laser according to the directivity of DFB fiber laser which is proved in the above experiments. Then three fiber lasers with different angle are used to get the location of the AE source. As shown in Figure 10, we can get three lines which intersect at A (x_4_, y_4_), B (x_5_, y_5_) and C (x_6_, y_6_). These points form a triangle ABC whose center of gravity is the location of AE source. The coordinate of the center of gravity, which is replaced by O (x, y), can be calculated by a simple triangle principle.

**Figure 11.**
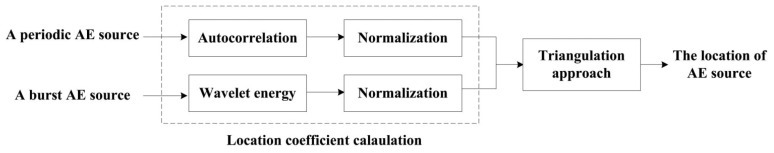
A specific location algorithm based on the directional sensitivity of DFB fiber laser and the triangulation approach.

We can thus determine the location of the AE source as we know the coordinates of O (x, y). Then a specific location algorithm based on the directional sensitivity of DFB fiber laser and the triangulation approach is shown in Figure 11. The advantage of the proposed algorithm over the traditional methods based on fiber Bragg Grating (FBG) include the capability of: having higher strain resolution for AE detection and taking into account two different types of AE source. The location calculate algorithm for two different types of AE source consists of three steps: (1) judge the type of the AE source; (2) calculate the location coefficient for AE source location and (3) use the triangulation approach to determine the location of AE source. Here, normalization is processed to eliminate the influence of distance and intensity of AE source.

## 5. Experiment and Results

**Figure 12.**
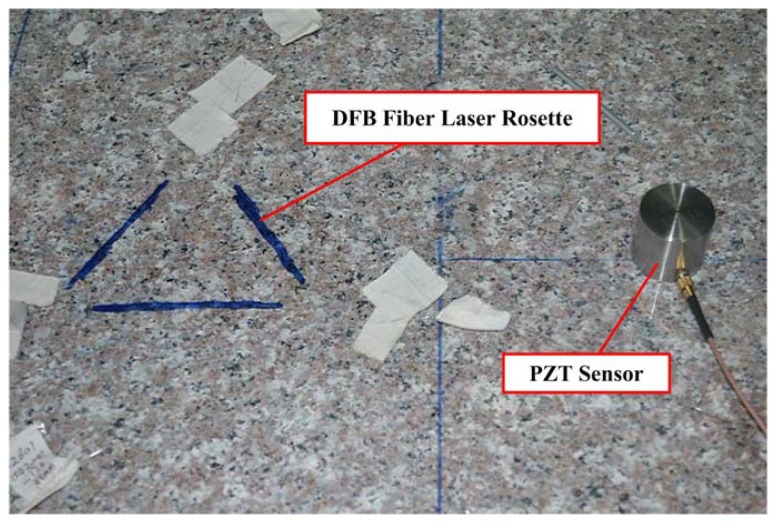
DFB fiber laser rosette for acoustic emission location.

Here, a Delta (Δ) rosette configuration based on three DFB fiber lasers is established according to the directivity of DFB fiber laser. The novel acoustic emission source location algorithm is used and tested to determine the direction and the location of acoustic emission waves. In this experiment, the DFB fiber laser rosette is glued to the marble plate using 353ND fiber adhesive. A PZT sensor is used as a continuous AE source and a steel ball is used as a low-velocity impact source, which is shown in Figure 12. The location experiment based on DFB fiber laser rosette is carried out by mounting PZT transducers or steel ball in turn on the marble plate in different places beyond the rosette. Then we can evaluate the location accuracy when the AE source is generated at different distances.

**Figure 13.**
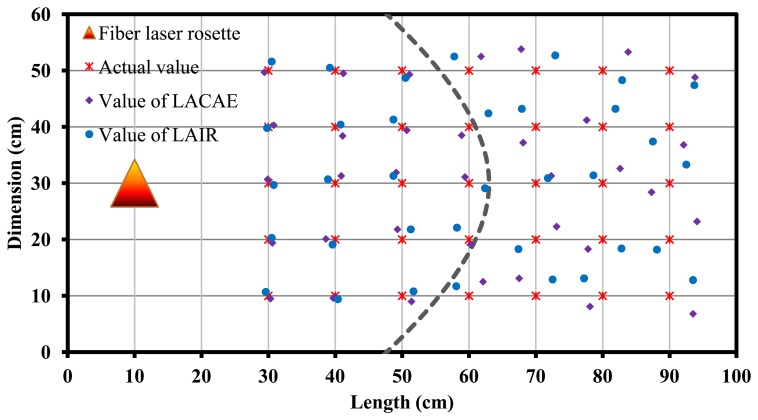
The results of location using DFB fiber laser rosette.

**Figure 14.**
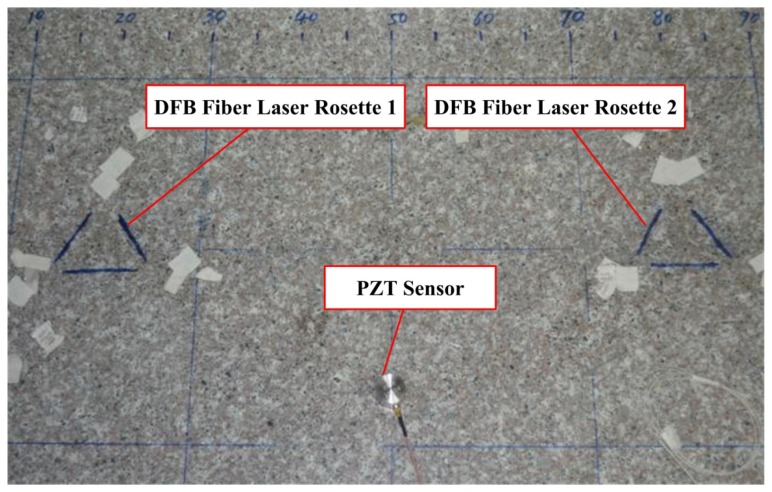
DFB fiber laser rosettes array for acoustic emission location.

**Figure 15.**
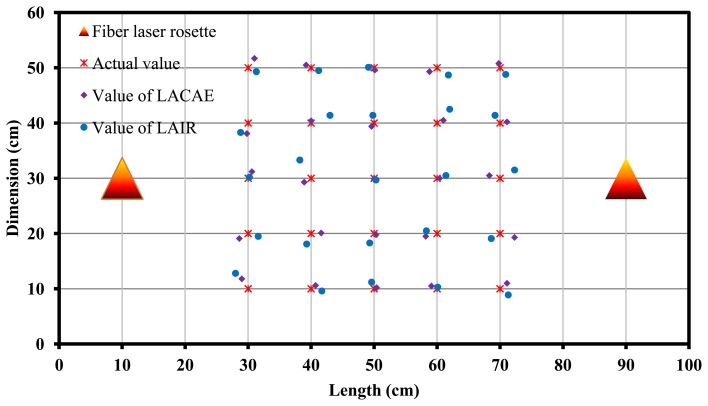
The results of location using a pair of DFB fiber laser rosettes.

The AE source location test based on one DFB fiber laser rosette (Figure 13) and two rosettes (Figures 14 and 15) were performed. From the test result, we can see that the location error is less than 2 cm within the circumference of 50 cm where the fiber laser rosette is the center circle. And the location accuracy and distance is limited by the distance because of the attenuation and interference of AE signals when the acoustic waves spread in the marble plate. The location algorithm of a continuous AE source (LACAE) is based on the relationship between wavelength drift and the angle of a continuous AE source. The location algorithm of an impact response (LAIR) is based on the relationship between percentage of wavelet packet energy and the angle of an impact response.

However, the experiment is conducted under the condition of quiet environment. In fact, the environmental vibration and temperature fluctuations may lead to a wrong result of AE source location. We can eliminate the influence of temperature fluctuations by digital filter processing, but the environmental vibration cannot be filtered simply by digital processing method. A specific packaging structure, which is immune to environmental strain, is required for engineering application in health monitoring of civil structures. A safety guard should be used to protect the DFB fiber laser because of the fragility of the fiber.

## 6. Conclusions

In this work, an application of DFB fiber lasers for AE source localization, based on a method of digital signal analysis, is presented and discussed. The proposed location algorithm has higher strain resolution for AE detection compared with the traditional methods based on fiber Bragg Grating (FBG) and can eliminate the influence of distance and intensity of AE source. Especially, we take into account two different types of AE source for location in this algorithm. This is the first demonstration, to the best of our knowledge, that a periodic AE signal or a burst AE source can be located by one location algorithm at the same time, providing a powerful technique for AE source location in health monitoring of civil structures.
